# [(*Z*)-*N*-(2-Chloro­phen­yl)-*O*-ethyl­thio­carbamato-κ*S*](triphenyl­phosphine-κ*P*)gold(I)

**DOI:** 10.1107/S1600536809049459

**Published:** 2009-11-25

**Authors:** Primjira P. Tadbuppa, Edward R. T. Tiekink

**Affiliations:** a Department of Chemistry, National University of Singapore, Singapore 117543; b Department of Chemistry, University of Malaya, 50603 Kuala Lumpur, Malaysia

## Abstract

The title compound, [Au(C_9_H_9_ClNOS)(C_18_H_15_P)], features a linear *S*,*P*-donor set with a small deviation from the ideal linear geometry due to the proximity of the meth­oxy O atom to Au [Au⋯O = 2.986 (4) Å].

## Related literature

For structural systematics and luminescence properties of phosphinegold(I) carbonimidothio­ates, see: Ho *et al.* (2006 ▶); Ho & Tiekink (2007 ▶); Kuan *et al.* (2008 ▶). For the synthesis, see Hall *et al.* (1993 ▶).
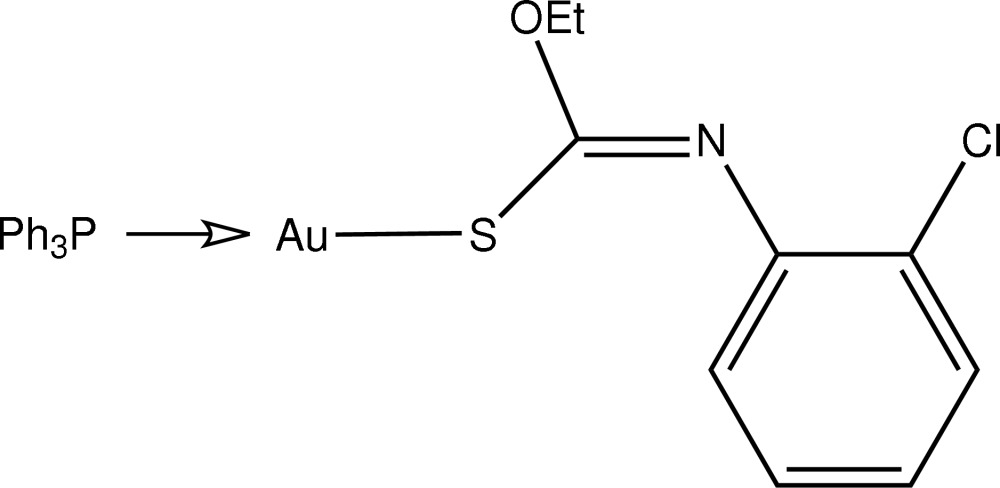


## Experimental

### 

#### Crystal data

[Au(C_9_H_9_ClNOS)(C_18_H_15_P)]
*M* *_r_* = 673.92
Monoclinic, 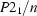

*a* = 8.8401 (5) Å
*b* = 26.0187 (14) Å
*c* = 10.8595 (6) Å
β = 94.850 (1)°
*V* = 2488.8 (2) Å^3^
*Z* = 4
Mo *K*α radiation
μ = 6.19 mm^−1^
*T* = 223 K
0.32 × 0.16 × 0.02 mm

#### Data collection

Bruker SMART CCD diffractometer
Absorption correction: multi-scan (*SADABS*; Bruker, 2000 ▶) *T*_min_ = 0.410, *T*_max_ = 1
17516 measured reflections
5712 independent reflections
4803 reflections with *I* > 2σ(*I*)
*R*_int_ = 0.049

#### Refinement

*R*[*F* ^2^ > 2σ(*F* ^2^)] = 0.035
*wR*(*F* ^2^) = 0.102
*S* = 1.03
5712 reflections
298 parameters
H-atom parameters constrained
Δρ_max_ = 1.69 e Å^−3^
Δρ_min_ = −0.98 e Å^−3^

### 

Data collection: *SMART* (Bruker, 2000 ▶); cell refinement: *SAINT* (Bruker, 2000 ▶); data reduction: *SHELXTL* (Sheldrick, 2008 ▶); program(s) used to solve structure: *PATTY* in *DIRDIF92* (Beurskens *et al.*, 1992 ▶); program(s) used to refine structure: *SHELXL97* (Sheldrick, 2008 ▶); molecular graphics: *DIAMOND* (Brandenburg, 2006 ▶); software used to prepare material for publication: *publCIF* (Westrip, 2009 ▶).

## Supplementary Material

Crystal structure: contains datablocks global, I. DOI: 10.1107/S1600536809049459/sj2690sup1.cif

Structure factors: contains datablocks I. DOI: 10.1107/S1600536809049459/sj2690Isup2.hkl

Additional supplementary materials:  crystallographic information; 3D view; checkCIF report

## Figures and Tables

**Table:** 

Au—S1	2.3131 (11)
Au—P1	2.2574 (11)

**Table:** 

S1—Au—P1	177.45 (4)

**Fig. 1.**
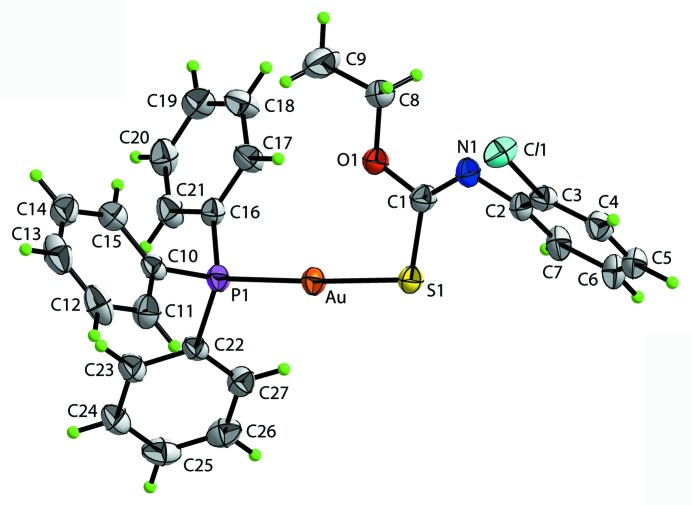
Molecular structure of (I) showing atom-labelling scheme and displacement ellipsoids at the 50% probability level.

## supplementary crystallographic information

### Comment

As a part of systematic studies of phosphinegold(I) thiocarbamides (Ho *et al.* 2006; Ho & Tiekink, 2007; Kuan *et al.*, 2008), the title compound, (C_5_H_5_)_3_PAu[SC(OEt)N(C_6_H_4_Cl-*o*)], was synthesized, (I). In (I), Fig. 1, the thiocarbamide functions as a thiolate ligand, a conclusion confirmed by the magnitudes of the C1—S1 and C1═N1 bond distances of 1.759 (5) and 1.265 (6) Å, respectively; the conformation about C1N1 is *Z*. A twist is evident between the central SC(O)N chromophore (maximum deviation = 0.021 (5) Å for the C1 atom) and the N-bound aryl ring as seen in the C1–N1–C2–C3 torsion angle of -133.7 (5)°. The thiocarbamato and phosphine ligands define an *S*, *P* donor set, Table 1. The deviation of the S1—Au—P1 angle [177.45 (4) °] from linearity is ascribed to the close approach of the O1 atom to Au [2.986 (4) Å].

### Experimental

Compound (I) was prepared following the standard literature procedure from the reaction of Ph_3_AuCl and EtOC(S)N(H)(C_6_H_4_Cl-*o*) in the presence of base (Hall *et al.*, 1993).

### Refinement

The H atoms were geometrically placed (C—H = 0.94–0.98 Å) and refined as riding with *U_iso_*(H) = 1.2–1.5*U*_eq_(C). A rotating group model was used for the methyl groups. The maximum and minimum residual electron density peaks of 1.69 and 0.98 e Å^-3^, respectively, were located 0.83 Å and 1.47 Å from the Au atom.

### Crystal data

**Table:**

[Au(C_9_H_9_ClNOS)(C_18_H_15_P)]	*F*(000) = 1312
*M**_r_* = 673.92	*D*_x_ = 1.799 Mg m^−^^3^
Monoclinic, *P*2_1_/*n*	Mo *K*α radiation, λ = 0.71069 Å
Hall symbol: -P 2yn	Cell parameters from 5468 reflections
*a* = 8.8401 (5) Å	θ = 2.5–28.2°
*b* = 26.0187 (14) Å	µ = 6.19 mm^−^^1^
*c* = 10.8595 (6) Å	*T* = 223 K
β = 94.850 (1)°	Plate, colourless
*V* = 2488.8 (2) Å^3^	0.32 × 0.16 × 0.02 mm
*Z* = 4	

### Data collection

**Table:**

Bruker SMART CCD diffractometer	5712 independent reflections
Radiation source: fine-focus sealed tube	4803 reflections with *I* > 2σ(*I*)
graphite	*R*_int_ = 0.049
ω scans	θ_max_ = 27.5°, θ_min_ = 1.6°
Absorption correction: multi-scan (*SADABS*; Bruker, 2000)	*h* = −9→11
*T*_min_ = 0.410, *T*_max_ = 1	*k* = −33→32
17516 measured reflections	*l* = −14→13

### Refinement

**Table:**

Refinement on *F*^2^	Primary atom site location: structure-invariant direct methods
Least-squares matrix: full	Secondary atom site location: difference Fourier map
*R*[*F*^2^ > 2σ(*F*^2^)] = 0.035	Hydrogen site location: inferred from neighbouring sites
*wR*(*F*^2^) = 0.102	H-atom parameters constrained
*S* = 1.03	*w* = 1/[σ^2^(*F*_o_^2^) + (0.0543*P*)^2^] where *P* = (*F*_o_^2^ + 2*F*_c_^2^)/3
5712 reflections	(Δ/σ)_max_ = 0.001
298 parameters	Δρ_max_ = 1.69 e Å^−^^3^
0 restraints	Δρ_min_ = −0.98 e Å^−^^3^

### Special details

**Table:**

Geometry. All s.u.'s (except the s.u. in the dihedral angle between two l.s. planes) are estimated using the full covariance matrix. The cell s.u.'s are taken into account individually in the estimation of s.u.'s in distances, angles and torsion angles; correlations between s.u.'s in cell parameters are only used when they are defined by crystal symmetry. An approximate (isotropic) treatment of cell s.u.'s is used for estimating s.u.'s involving l.s. planes.
Refinement. Refinement of *F*^2^ against ALL reflections. The weighted *R*-factor *wR* and goodness of fit *S* are based on *F*^2^, conventional *R*-factors *R* are based on *F*, with *F* set to zero for negative *F*^2^. The threshold expression of *F*^2^ > 2σ(*F*^2^) is used only for calculating *R*-factors(gt) *etc*. and is not relevant to the choice of reflections for refinement. *R*-factors based on *F*^2^ are statistically about twice as large as those based on *F*, and *R*- factors based on ALL data will be even larger.

### Fractional atomic coordinates and isotropic or equivalent isotropic displacement parameters (Å^2^)

**Table:**

	*x*	*y*	*z*	*U*_iso_*/*U*_eq_	
Au	0.239069 (19)	0.104287 (7)	0.092536 (15)	0.03276 (8)	
Cl1	0.91579 (15)	0.17981 (6)	−0.11081 (11)	0.0464 (3)	
S1	0.36556 (14)	0.14702 (5)	−0.05484 (10)	0.0355 (3)	
P1	0.12476 (13)	0.06311 (5)	0.24242 (10)	0.0306 (3)	
O1	0.5141 (4)	0.16861 (14)	0.1570 (3)	0.0367 (8)	
N1	0.6231 (5)	0.20353 (17)	−0.0042 (4)	0.0376 (9)	
C1	0.5152 (5)	0.1776 (2)	0.0337 (4)	0.0344 (10)	
C2	0.6273 (5)	0.2179 (2)	−0.1288 (4)	0.0351 (10)	
C3	0.7614 (5)	0.21131 (19)	−0.1871 (4)	0.0337 (10)	
C4	0.7746 (6)	0.2294 (2)	−0.3057 (4)	0.0405 (11)	
H4	0.8658	0.2246	−0.3429	0.049*	
C5	0.6550 (6)	0.2542 (2)	−0.3693 (5)	0.0429 (12)	
H5	0.6635	0.2663	−0.4500	0.051*	
C6	0.5227 (6)	0.2612 (2)	−0.3132 (5)	0.0440 (12)	
H6	0.4403	0.2781	−0.3563	0.053*	
C7	0.5093 (6)	0.2439 (2)	−0.1948 (5)	0.0418 (12)	
H7	0.4184	0.2497	−0.1579	0.050*	
C8	0.6417 (6)	0.1891 (2)	0.2348 (5)	0.0462 (13)	
H8A	0.6378	0.2267	0.2348	0.055*	
H8B	0.7373	0.1784	0.2029	0.055*	
C9	0.6343 (8)	0.1697 (3)	0.3603 (5)	0.073 (2)	
H9A	0.7201	0.1826	0.4128	0.110*	
H9B	0.6371	0.1324	0.3595	0.110*	
H9C	0.5406	0.1811	0.3921	0.110*	
C10	0.2264 (5)	0.00525 (19)	0.2929 (4)	0.0328 (10)	
C11	0.2483 (6)	−0.0325 (2)	0.2058 (5)	0.0456 (13)	
H11	0.2094	−0.0276	0.1233	0.055*	
C12	0.3266 (7)	−0.0772 (2)	0.2384 (6)	0.0520 (14)	
H12	0.3398	−0.1025	0.1784	0.062*	
C13	0.3852 (7)	−0.0848 (2)	0.3588 (7)	0.0558 (16)	
H13	0.4391	−0.1150	0.3809	0.067*	
C14	0.3644 (6)	−0.0479 (2)	0.4462 (6)	0.0526 (15)	
H14	0.4027	−0.0532	0.5286	0.063*	
C15	0.2867 (6)	−0.0026 (2)	0.4131 (5)	0.0442 (13)	
H15	0.2752	0.0228	0.4732	0.053*	
C16	0.1206 (6)	0.10257 (17)	0.3797 (4)	0.0319 (10)	
C17	0.2334 (6)	0.1390 (2)	0.4037 (5)	0.0439 (12)	
H17	0.3034	0.1450	0.3448	0.053*	
C18	0.2451 (7)	0.1666 (2)	0.5129 (5)	0.0498 (14)	
H18	0.3229	0.1910	0.5285	0.060*	
C19	0.1414 (7)	0.1580 (2)	0.5989 (5)	0.0465 (13)	
H19	0.1498	0.1760	0.6742	0.056*	
C20	0.0255 (7)	0.1231 (2)	0.5749 (5)	0.0465 (13)	
H20	−0.0474	0.1184	0.6320	0.056*	
C21	0.0165 (7)	0.0948 (2)	0.4661 (5)	0.0443 (13)	
H21	−0.0607	0.0702	0.4511	0.053*	
C22	−0.0695 (5)	0.04190 (18)	0.2042 (4)	0.0311 (9)	
C23	−0.1225 (6)	−0.0036 (2)	0.2526 (5)	0.0455 (13)	
H23	−0.0565	−0.0245	0.3032	0.055*	
C24	−0.2714 (6)	−0.0178 (3)	0.2264 (5)	0.0538 (15)	
H24	−0.3072	−0.0485	0.2595	0.065*	
C25	−0.3683 (6)	0.0124 (3)	0.1522 (5)	0.0506 (14)	
H25	−0.4700	0.0025	0.1346	0.061*	
C26	−0.3165 (6)	0.0573 (2)	0.1034 (5)	0.0472 (13)	
H26	−0.3831	0.0781	0.0531	0.057*	
C27	−0.1665 (6)	0.0718 (2)	0.1284 (5)	0.0389 (11)	
H27	−0.1307	0.1021	0.0937	0.047*	

### Atomic displacement parameters (Å^2^)

**Table:**

	*U*^11^	*U*^22^	*U*^33^	*U*^12^	*U*^13^	*U*^23^
Au	0.03321 (12)	0.03459 (13)	0.03128 (12)	−0.00426 (7)	0.00752 (8)	0.00257 (7)
Cl1	0.0390 (6)	0.0635 (9)	0.0364 (6)	0.0048 (6)	0.0010 (5)	0.0004 (6)
S1	0.0370 (6)	0.0421 (7)	0.0278 (5)	−0.0071 (5)	0.0054 (4)	0.0029 (5)
P1	0.0301 (6)	0.0316 (6)	0.0307 (5)	−0.0039 (5)	0.0064 (5)	0.0022 (5)
O1	0.0388 (18)	0.043 (2)	0.0284 (15)	−0.0089 (15)	0.0051 (14)	0.0008 (14)
N1	0.038 (2)	0.041 (2)	0.0341 (19)	−0.0061 (18)	0.0068 (17)	0.0066 (18)
C1	0.036 (2)	0.040 (3)	0.028 (2)	−0.001 (2)	0.0068 (19)	−0.001 (2)
C2	0.036 (2)	0.038 (3)	0.032 (2)	−0.006 (2)	0.0055 (19)	0.006 (2)
C3	0.031 (2)	0.036 (3)	0.034 (2)	−0.0022 (19)	0.0021 (18)	−0.002 (2)
C4	0.043 (3)	0.042 (3)	0.039 (2)	−0.007 (2)	0.010 (2)	−0.001 (2)
C5	0.053 (3)	0.039 (3)	0.037 (2)	−0.005 (2)	0.004 (2)	0.008 (2)
C6	0.052 (3)	0.035 (3)	0.045 (3)	0.002 (2)	0.005 (2)	0.013 (2)
C7	0.040 (3)	0.037 (3)	0.050 (3)	0.000 (2)	0.011 (2)	0.011 (2)
C8	0.049 (3)	0.050 (3)	0.038 (3)	−0.011 (3)	−0.003 (2)	0.002 (2)
C9	0.070 (5)	0.110 (6)	0.040 (3)	−0.031 (4)	−0.003 (3)	0.007 (4)
C10	0.028 (2)	0.033 (3)	0.037 (2)	−0.0068 (18)	0.0070 (18)	0.001 (2)
C11	0.049 (3)	0.045 (3)	0.043 (3)	0.002 (2)	0.010 (2)	0.004 (2)
C12	0.056 (3)	0.029 (3)	0.074 (4)	0.000 (2)	0.025 (3)	−0.002 (3)
C13	0.040 (3)	0.041 (3)	0.087 (5)	0.004 (2)	0.008 (3)	0.016 (3)
C14	0.046 (3)	0.049 (4)	0.060 (3)	−0.003 (3)	−0.009 (3)	0.018 (3)
C15	0.047 (3)	0.040 (3)	0.045 (3)	−0.007 (2)	−0.002 (2)	0.002 (2)
C16	0.036 (2)	0.028 (2)	0.033 (2)	0.0018 (18)	0.0050 (19)	0.0028 (18)
C17	0.045 (3)	0.041 (3)	0.047 (3)	−0.012 (2)	0.010 (2)	−0.006 (2)
C18	0.051 (3)	0.046 (3)	0.053 (3)	−0.012 (3)	0.004 (3)	−0.015 (3)
C19	0.057 (3)	0.041 (3)	0.042 (3)	0.010 (3)	0.006 (2)	−0.008 (2)
C20	0.059 (3)	0.041 (3)	0.043 (3)	0.002 (3)	0.024 (3)	0.000 (2)
C21	0.048 (3)	0.042 (3)	0.045 (3)	−0.007 (2)	0.018 (2)	0.000 (2)
C22	0.030 (2)	0.031 (2)	0.033 (2)	−0.0043 (18)	0.0058 (18)	−0.0025 (19)
C23	0.039 (3)	0.045 (3)	0.052 (3)	−0.011 (2)	−0.002 (2)	0.011 (3)
C24	0.046 (3)	0.055 (4)	0.060 (3)	−0.020 (3)	0.001 (3)	0.011 (3)
C25	0.031 (3)	0.068 (4)	0.053 (3)	−0.008 (3)	0.003 (2)	−0.012 (3)
C26	0.040 (3)	0.052 (3)	0.048 (3)	0.011 (3)	−0.005 (2)	−0.002 (3)
C27	0.040 (3)	0.036 (3)	0.040 (2)	0.005 (2)	0.003 (2)	0.000 (2)

### Geometric parameters (Å, °)

**Table:**

Au—S1	2.3131 (11)	C11—H11	0.9400
Au—P1	2.2574 (11)	C12—C13	1.379 (9)
Cl1—C3	1.740 (5)	C12—H12	0.9400
S1—C1	1.759 (5)	C13—C14	1.372 (9)
P1—C16	1.813 (5)	C13—H13	0.9400
P1—C10	1.814 (5)	C14—C15	1.396 (8)
P1—C22	1.818 (5)	C14—H14	0.9400
O1—C1	1.360 (5)	C15—H15	0.9400
O1—C8	1.453 (6)	C16—C17	1.384 (7)
N1—C1	1.265 (6)	C16—C21	1.384 (7)
N1—C2	1.407 (6)	C17—C18	1.383 (7)
C2—C7	1.391 (7)	C17—H17	0.9400
C2—C3	1.401 (6)	C18—C19	1.382 (8)
C3—C4	1.385 (7)	C18—H18	0.9400
C4—C5	1.375 (7)	C19—C20	1.377 (8)
C4—H4	0.9400	C19—H19	0.9400
C5—C6	1.376 (7)	C20—C21	1.388 (8)
C5—H5	0.9400	C20—H20	0.9400
C6—C7	1.377 (7)	C21—H21	0.9400
C6—H6	0.9400	C22—C27	1.378 (7)
C7—H7	0.9400	C22—C23	1.392 (7)
C8—C9	1.460 (7)	C23—C24	1.374 (7)
C8—H8A	0.9800	C23—H23	0.9400
C8—H8B	0.9800	C24—C25	1.374 (8)
C9—H9A	0.9700	C24—H24	0.9400
C9—H9B	0.9700	C25—C26	1.376 (9)
C9—H9C	0.9700	C25—H25	0.9400
C10—C15	1.383 (7)	C26—C27	1.384 (8)
C10—C11	1.389 (7)	C26—H26	0.9400
C11—C12	1.384 (8)	C27—H27	0.9400
			
S1—Au—P1	177.45 (4)	C13—C12—C11	120.1 (6)
C1—S1—Au	102.98 (16)	C13—C12—H12	120.0
C16—P1—C10	105.4 (2)	C11—C12—H12	120.0
C16—P1—C22	106.0 (2)	C14—C13—C12	119.7 (6)
C10—P1—C22	104.5 (2)	C14—C13—H13	120.2
C16—P1—Au	111.44 (16)	C12—C13—H13	120.2
C10—P1—Au	111.79 (15)	C13—C14—C15	120.3 (5)
C22—P1—Au	116.84 (15)	C13—C14—H14	119.9
C1—O1—C8	116.1 (4)	C15—C14—H14	119.9
C1—N1—C2	122.4 (4)	C10—C15—C14	120.6 (5)
N1—C1—O1	118.8 (4)	C10—C15—H15	119.7
N1—C1—S1	128.1 (4)	C14—C15—H15	119.7
O1—C1—S1	113.1 (3)	C17—C16—C21	118.8 (5)
C7—C2—C3	116.9 (4)	C17—C16—P1	118.6 (4)
C7—C2—N1	123.0 (4)	C21—C16—P1	122.4 (4)
C3—C2—N1	119.7 (4)	C18—C17—C16	121.2 (5)
C4—C3—C2	121.4 (5)	C18—C17—H17	119.4
C4—C3—Cl1	118.6 (4)	C16—C17—H17	119.4
C2—C3—Cl1	120.1 (4)	C19—C18—C17	119.3 (5)
C5—C4—C3	120.3 (5)	C19—C18—H18	120.4
C5—C4—H4	119.8	C17—C18—H18	120.4
C3—C4—H4	119.8	C20—C19—C18	120.3 (5)
C4—C5—C6	119.1 (5)	C20—C19—H19	119.9
C4—C5—H5	120.4	C18—C19—H19	119.9
C6—C5—H5	120.4	C19—C20—C21	120.0 (5)
C5—C6—C7	120.9 (5)	C19—C20—H20	120.0
C5—C6—H6	119.6	C21—C20—H20	120.0
C7—C6—H6	119.6	C16—C21—C20	120.4 (5)
C6—C7—C2	121.4 (5)	C16—C21—H21	119.8
C6—C7—H7	119.3	C20—C21—H21	119.8
C2—C7—H7	119.3	C27—C22—C23	119.6 (4)
O1—C8—C9	108.8 (5)	C27—C22—P1	119.5 (4)
O1—C8—H8A	109.9	C23—C22—P1	120.9 (4)
C9—C8—H8A	109.9	C24—C23—C22	119.9 (5)
O1—C8—H8B	109.9	C24—C23—H23	120.1
C9—C8—H8B	109.9	C22—C23—H23	120.1
H8A—C8—H8B	108.3	C25—C24—C23	120.4 (5)
C8—C9—H9A	109.5	C25—C24—H24	119.8
C8—C9—H9B	109.5	C23—C24—H24	119.8
H9A—C9—H9B	109.5	C24—C25—C26	120.1 (5)
C8—C9—H9C	109.5	C24—C25—H25	120.0
H9A—C9—H9C	109.5	C26—C25—H25	120.0
H9B—C9—H9C	109.5	C25—C26—C27	120.0 (5)
C15—C10—C11	118.3 (5)	C25—C26—H26	120.0
C15—C10—P1	123.3 (4)	C27—C26—H26	120.0
C11—C10—P1	118.4 (4)	C22—C27—C26	120.0 (5)
C12—C11—C10	121.1 (5)	C22—C27—H27	120.0
C12—C11—H11	119.5	C26—C27—H27	120.0
C10—C11—H11	119.5		
			
C2—N1—C1—O1	−175.2 (4)	C11—C10—C15—C14	−1.4 (8)
C2—N1—C1—S1	9.1 (8)	P1—C10—C15—C14	−179.7 (4)
C8—O1—C1—N1	−1.4 (7)	C13—C14—C15—C10	1.6 (9)
C8—O1—C1—S1	174.9 (4)	C10—P1—C16—C17	93.6 (4)
Au—S1—C1—N1	175.3 (5)	C22—P1—C16—C17	−155.9 (4)
Au—S1—C1—O1	−0.6 (4)	Au—P1—C16—C17	−27.8 (5)
C1—N1—C2—C7	53.7 (8)	C10—P1—C16—C21	−81.0 (5)
C1—N1—C2—C3	−133.7 (5)	C22—P1—C16—C21	29.5 (5)
C7—C2—C3—C4	−0.8 (7)	Au—P1—C16—C21	157.6 (4)
N1—C2—C3—C4	−173.8 (5)	C21—C16—C17—C18	1.3 (8)
C7—C2—C3—Cl1	179.3 (4)	P1—C16—C17—C18	−173.5 (4)
N1—C2—C3—Cl1	6.4 (7)	C16—C17—C18—C19	−0.6 (9)
C2—C3—C4—C5	−0.1 (8)	C17—C18—C19—C20	−1.4 (9)
Cl1—C3—C4—C5	179.7 (4)	C18—C19—C20—C21	2.8 (9)
C3—C4—C5—C6	0.5 (8)	C17—C16—C21—C20	0.0 (8)
C4—C5—C6—C7	0.2 (8)	P1—C16—C21—C20	174.6 (4)
C5—C6—C7—C2	−1.2 (9)	C19—C20—C21—C16	−2.0 (9)
C3—C2—C7—C6	1.5 (8)	C16—P1—C22—C27	88.7 (4)
N1—C2—C7—C6	174.2 (5)	C10—P1—C22—C27	−160.2 (4)
C1—O1—C8—C9	−171.8 (5)	Au—P1—C22—C27	−36.1 (4)
C16—P1—C10—C15	−1.3 (5)	C16—P1—C22—C23	−89.9 (4)
C22—P1—C10—C15	−112.8 (4)	C10—P1—C22—C23	21.1 (5)
Au—P1—C10—C15	119.9 (4)	Au—P1—C22—C23	145.3 (4)
C16—P1—C10—C11	−179.6 (4)	C27—C22—C23—C24	−1.2 (8)
C22—P1—C10—C11	68.9 (4)	P1—C22—C23—C24	177.4 (5)
Au—P1—C10—C11	−58.4 (4)	C22—C23—C24—C25	0.3 (9)
C15—C10—C11—C12	0.9 (8)	C23—C24—C25—C26	0.1 (9)
P1—C10—C11—C12	179.2 (4)	C24—C25—C26—C27	0.4 (9)
C10—C11—C12—C13	−0.4 (9)	C23—C22—C27—C26	1.7 (8)
C11—C12—C13—C14	0.5 (9)	P1—C22—C27—C26	−177.0 (4)
C12—C13—C14—C15	−1.1 (9)	C25—C26—C27—C22	−1.3 (8)
